# Supplementary figures and images for: Alternative Splicing for Leucanthemella linearis NST1 Contributes to Variable Abiotic Stress Resistance in Transgenic Tobacco
Source: Genes (Basel). 2023 Jul 28;14(8):1549. doi: 10.3390/genes14081549 (PMC10454811; doi:10.3390/genes14081549)

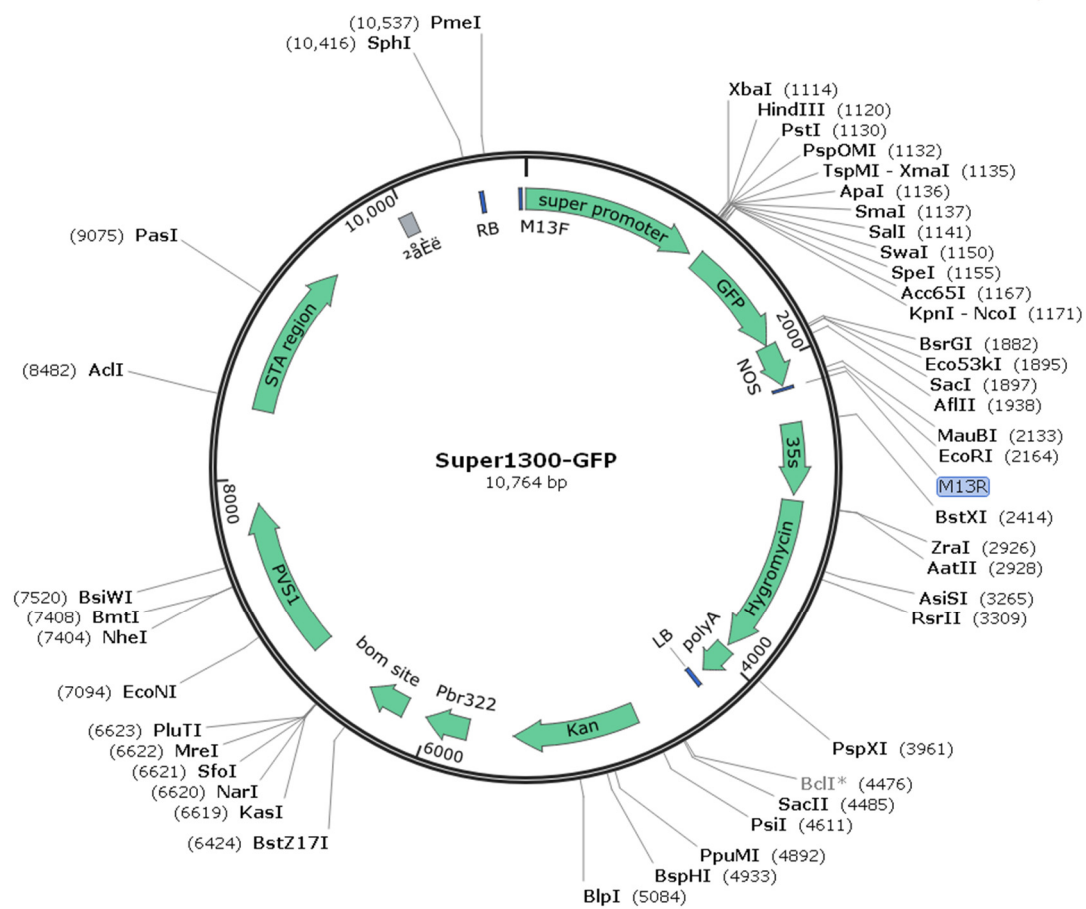

Supplement: Supplementary file 1 [file genes-14-01549-s001.zip › Figure S1super35S carrier structure.pdf]
